# Sex‐related association of modifiable risk factors with hypertension: A national cross‐sectional study of NHANES 2007–2018

**DOI:** 10.1002/clc.24165

**Published:** 2023-10-05

**Authors:** Jingya Niu, Demin Xu, Yujie Huang, Jianhong You, Jie Zhang, Jianan Li, Dan Su, Sanru Lin, Lixia Suo, Jianying Ma, Shujing Wu

**Affiliations:** ^1^ Jiading District Central Hospital Affiliated Shanghai University of Medicine and Health Sciences Shanghai China; ^2^ School of Clinical Medicine Shanghai University of Medicine and Health Sciences Shanghai China; ^3^ Department of Cardiac Surgery, Zhongshan Hospital Fudan University Shanghai China; ^4^ Medical Department, Zhongshan Hospital (Xiamen) Fudan University Xiamen Fujian China; ^5^ Department of Ultrasound, Zhongshan Hospital of Xiamen University, School of Medicine Xiamen University Xiamen Fujian China; ^6^ School of Public Health Xiamen University Xiamen Fujian China; ^7^ Department of Cardiology, Zhongshan Hospital (Xiamen) Fudan University Xiamen Fujian China; ^8^ Department of Cardiology, Zhongshan Hospital Fudan University Shanghai China

**Keywords:** hypertension, population attributable fraction, risk factors, sex

## Abstract

**Objective:**

Sex difference is commonly observed in hypertension. We aimed to assess sex differences in the associations of modifiable lifestyle and metabolic risk factors with risk of hypertension.

**Design:**

National cross‐sectional population study.

**Setting:**

Data from the 2007 to 2018 National Health and Nutrition Examination Survey.

**Participants:**

7087 adults aged ≥30 years without a prior history of hypertension.

**Primary and Secondary Outcome Measures:**

Odds ratios and population attributable fraction (PAF) of hypertension associated with 10 modifiable risk factors: five lifestyle risk factors (current smoking, excess alcohol intake, poor diet, physical inactivity, and unhealthy sleep), and five metabolic risk factors (obesity, diabetes, dyslipidaemia, hyperuricemia, and chronic kidney disease) in women versus men.

**Results:**

Compared with women, men had 84% increased risk of prevalence of hypertension. The sex difference in risk for hypertension is more evident in those aged <60 years (*p* for interaction <.001). For those aged <60 years the combination of lifestyle risk factors accounted for a PAF of 27.2% in men and 48.8% in women, and the combination of metabolic risk factors accounted for a PAF similarly in men (37.4%) and women (38.2%). For those aged ≥60 years, the PAF of lifestyle risk factors was similar between men and women and the metabolic risk factors accounted for a greater proportion in women (33.0% vs. 14.5% in men).

**Conclusions:**

Sex differences may exist in the relation and attribution of lifestyle and metabolic risk factors to hypertension, which may have implications for implementing sex‐specific strategies to prevent hypertension.

## Graphical abstract

This study included 7087 participants aged ≥30 years from NHANES and showed that men had 84% increased risk of prevalence of hypertension than women. The sex difference was more evident in those aged <60 years and lifestyle and metabolic risk factors accounted for different attribution to hypertension between men and women.

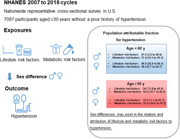

## 1 INTRODUCTION

Hypertension is a leading cause of morbidity and mortality worldwide.^1^ Blood pressure (BP) always increases with age, while the patterns of increase diverge in males and females.^2^ Sex differences is commonly observed in the prevalence, therapy, and prognosis of hypertension.^3^, ^4^, ^5^

The 2017 American College of Cardiology (ACC) and the American Heart Association (AHA) hypertension guideline has updated the definition of hypertension as a BP level ≥130/80 mmHg.^6^ Emerging evidence have identified multiple modifiable risk factors for hypertension, such as smoking, excess alcohol consumption, poor diet, lack of physical activity, unhealthy sleep, obesity, diabetes, dyslipidemia, hyperuricemia, and chronic kidney disease (CKD).^7^, ^8^, ^9^, ^10^ However, these findings suggested that important sex differences in lifestyle and metabolic risk factors might exist among patients with hypertension.^11^, ^12^, ^13^ Therefore, identification of sex‐specific modifiable risk factor can help the development of personalized strategies for the prevention of hypertension.

Using data from the U.S. National Health and Nutrition Examination Survey (NHANES), we sought to examine whether sex modified the effect of common modifiable risk factors on hypertension. The associations and population‐attributable fractions were reported according to sex and age strata.

## 2 METHODS

### 2.1 Study population

NHANES is a nationwide representative cross‐sectional survey using a complex, multistage probability design. Details of the study design and data collection have been described by the National Center for Health Statistics (https://www.cdc.gov/nchs/nhanes/about_nhanes.htm). Our study included data from the NHANES 2007 to 2018 cycles.

**Figure 1:**
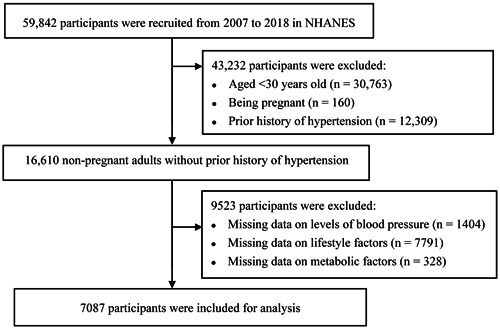
Flow chart of selecting subjects using National Health and Nutrition Examination Survey (NHANES) 2007–2018.

After excluding participants aged <30 years old (*n* = 30 763), being pregnant (*n* = 160) with prior self‐reported history of hypertension or using antihypertensive medications (*n* = 12 309), and those missing data on BP (*n* = 1404), lifestyle factors (*n* = 7791), and metabolic factors (*n* = 328), 7087 participants aged ≥30 years with complete and reliable information (demographics, health behaviors, body measurements, and disease information) were included (Figure 1).

### 2.2 Demographic variables

Age, sex, race, and education attainment were obtained through questionnaires. Race were classified into four categories: non‐Hispanic White, non‐Hispanic Black, Mexican American, and other race. Education level was divided into two levels: without high school diploma and high school education or above.

### 2.3 Lifestyle factors

A total of five lifestyle factors examined in this study included smoking status, alcohol consumption, dietary quality, physical activity, and sleep duration, which were obtained through questionnaires and 24 h dietary recalls. Participants who had more than 100 cigarettes in life and smoked some days or every day were defined as current smokers.^14^ Participants who had ≥2 drinks per day for females or ≥3 drinks per day for males were classified as excess alcohol intake.^14^ Dietary quality was assessed by healthy eating index (HEI) scores (scores ranged from 0 to 100, with higher HEI scores reflecting better diet quality) that measures the adherence to the 2015 Dietary Guidelines for Americans.^15^ A poor diet was defined as the HEI 2015 scores at the bottom three fourth of distribution. The metabolic equivalent (MET) was calculated to evaluate the leisure time physical activity. Physical inactivity was defined as <600 MET‐min per week.^16^ Sleep durations of <7 and >9 h per day at night were considered unhealthy.^17^

### 2.4 Metabolic factors

We selected five metabolic factors including obesity, diabetes, dyslipidaemia, hyperuricemia, and CKD, which were measured at mobile examination center (MEC) by trained technicians or obtained from laboratory data. Body mass index (BMI) was calculated as weight divided by height squared (kg/m^2^). We defined obesity as BMI ≥30 kg/m^2^.^18^ According to the 2010 American Diabetes Association criteria,^19^ diabetes was defined as a fasting glucose ≥126 mg/dL, random glucose or 2 h plasma glucose ≥200 mg/dL, HbA1c ≥6.5%, self‐reported history of diabetes, and/or using hypoglycemic drugs. Dyslipidemia was defined as total cholesterol ≥200 mg/dL, triglycerides (TG) ≥150 mg/dL, low‐density lipoprotein cholesterol (LDL‐C) ≥130 mg/dL, high‐density lipoprotein cholesterol (HDL‐C) <40 mg/dL (men) or HDL‐C <50 mg/dL (women), and/or using lipid‐lowering medications.^20^ The definition of hyperuricemia was a serum urate level >7.0 mg/dL among men and a serum urate level >5.7 mg/dL among women.^21^ The estimated glomerular filtration rate (eGFR) was calculated using the Chronic Kidney Disease‐Epidemiology Collaboration (CKD‐EPI) formula.^22^ CKD was defined by eGFR <60 mL/min/1.73 m^2^ or urinary albumin to creatinine ratio (UACR) ≥30 mg/g.^23^

### 2.5 Hypertension ascertainment

BP measurements were taken at MEC during the NHANES examination visits. After resting quietly in a sitting position for 5 min, BP was measured three times and a fourth attempt may be made if required. The average readings of systolic BP (SBP) and diastolic BP (DBP) were used for analysis. According to the 2017 ACC/AHA hypertension guideline,^6^ hypertension was defined as SBP ≥130 mmHg and (or) DBP ≥80 mmHg.

### 2.6 Statistical analyses

The characteristics of the participants by sex were described as weighted means, medians, and percentages for normally distributed data, skewed distributed variables, and categorical data, respectively. According to the NHANES statistical analysis guideline,^24^ survey weights from the MEC (exam weight) were adjusted for the combined NHANES cycles and the current analysis used survey strata and clusters to accommodate complex sampling in the weighted proportion calculations.

Weighted logistic regression was used to estimate odds ratios (ORs) and 95% confidence intervals (CIs) of hypertension in women versus men, either unadjusted or adjusted for covariates including age (continuous), race (White, Black, and other ethnic groups), education (< or ≥high school), current smoker (yes or no), excess alcohol intake (yes or no), healthy diet (yes or no), active physical activity (yes or no), healthy sleep duration (yes or no), obesity (yes or no), diabetes (yes or no), dyslipidemia (yes or no), hyperuricemia (yes or no), and CKD (yes or no). These analyses were repeated in adults aged <60 and ≥60 years separately. Interactions between age groups (< or ≥60 years) and sex in the association with risks of hypertension were estimated by including the product term in the models.

Each lifestyle factor was assigned 1 point for an unhealthy level and 0 points for a healthy level. Thus, the lifestyle and metabolic risk scores were the sum of individual component point, which ranged from 0 to 5 separately. We also evaluated the associations of individual risk factor and per 1‐point increment in risk scores with hypertension among participants aged ≥60 or <60 years by sex using weighted logistic models, adjusted for covariates including age (continuous), race (White, Black, and other ethnic groups), education (< or ≥high school). In addition, sensitivity analysis was conducted using multiple random forest imputations of missing data including lifestyle and metabolic risk factors.

To further quantified the effect of these risk factors on hypertension by sex and age, the population attributable fraction (PAF) and 95% CI of each risk factor was calculated with adjustment for age (continuous), race (White, Black, and other ethnic groups), education (< or ≥high school). The PAF is formulated as a function of relative risk(s) and the prevalence(s) of the risk factor(s) and calculated by the method described in detail by Spiegelman et al.^25^ Individual risk factor with a negative PAF was truncated at the value of point estimate of 0 and was excluded in the analyses of cumulative PAF of lifestyle and metabolic risk factors.

All the tests were two tailed, with a *p* < .05 considered to indicate statistical significance. SAS 9.4 and R version 4.1.3 were used for the statistical analyses.

## 3 RESULTS

Baseline characteristics of the overall 7087 participants (55.4% men) according to sex group are presented in Table 1. Compared with women, men had increased proportions of current smoker and poor diet; higher levels of BMI, fasting glucose, HbA1c, TG, LDL‐C, and serum uric acid; and lower levels of eGFR and UACR (all *p* values for trend <.05). Table S1 of the supplement showed the prevalence of the 10 modifiable risk factors of hypertension by age and sex categories.

**Table 1:** Characteristics of participants by sex group.

Characteristics	Overall *N* = 7087	Men *N* = 3927 (55.4%)	Women *N* = 3160 (44.6%)	*p* Value
Age (years)	46.00 (38.00–56.00)	46.00 (37.00–56.00)	47.00 (38.00–56.00)	.16
Race/ethnicity, *n* (%)				<.001
Non‐Hispanic White	73.99 (67.30–80.68)	71.19 (68.52–73.86)	77.09 (74.46–79.73)	
Non‐Hispanic Black	7.27 (6.43–8.12)	7.28 (6.24–8.32)	7.27 (6.10–8.45)	
Mexican American	7.73 (6.47–8.99)	9.45 (7.85–11.05)	5.83 (4.61–7.05)	
Others	11.00 (9.80–12.21)	12.08 (10.48–13.69)	9.81 (8.30–11.32)	
High school education or above, *n* (%)	89.99 (83.60–96.38)	87.74 (86.30–89.17)	92.49 (91.08–93.90)	<.001
Lifestyle factors				
Current smokers, *n* (%)	19.68 (18.25–21.11)	21.76 (19.92–23.61)	17.39 (15.58–19.19)	<.001
Excess alcohol intake, *n* (%)	48.95 (45.46–52.44)	44.08 (41.33–46.84)	54.33 (51.75–56.91)	<.001
Poor diet, *n* (%)	74.41 (69.75–79.07)	77.92 (76.09–79.75)	70.53 (68.00–73.06)	<.001
Physical inactivity, *n* (%)	68.11 (63.12–73.10)	60.15 (57.94–62.37)	76.91 (74.98–78.84)	<.001
Unhealthy sleep, *n* (%)	57.86 (53.66–62.07)	58.81 (56.25–61.38)	56.81 (53.47–60.16)	.17
Metabolic factors				
Obesity, *n* (%)	29.91 (27.70–32.12)	30.90 (28.73–33.07)	28.82 (26.84–30.80)	.15
BMI (kg/m^2^)	27.97 (27.75–28.18)	28.33 (28.08–28.58)	27.56 (27.25–27.87)	<.001
Diabetes, *n* (%)	7.01 (6.13–7.89)	8.43 (7.08–9.78)	5.44 (4.52–6.35)	<.001
Fasting glucose (mg/dL)	102.37 (101.51–103.23)	105.17 (103.99–106.35)	99.11 (98.14–100.08)	<.001
2 h OGTT glucose (mg/dL)	109.42 (107.47–111.37)	110.19 (107.17–113.21)	108.52 (105.82–111.21)	.44
HbA1c (%)	5.49 (5.47–5.51)	5.54 (5.50–5.57)	5.44 (5.42–5.46)	<.001
Dyslipidaemia, *n* (%)	68.86 (63.85–73.88)	70.36 (68.29–72.43)	67.21 (64.90–69.53)	.05
Triglycerides (mg/dL)	95.00 (65.00–144.00)	106.00 (72.00–156.00)	86.00 (60.00–125.00)	<.001
Total cholesterol (mg/dL)	200.86 (199.33–202.38)	199.98 (197.82–202.14)	201.82 (199.91–203.74)	.19
LDL cholesterol (mg/dL)	119.55 (118.02–121.08)	121.26 (119.21–123.31)	117.60 (115.51–119.70)	.01
HDL cholesterol (mg/dL)	55.98 (55.25–56.71)	49.67 (48.94–50.40)	62.97 (62.05–63.88)	<.001
Hyperuricemia, *n* (%)	14.90 (13.22–16.58)	17.17 (15.43–18.91)	12.39 (10.86–13.92)	<.001
Uric acid (mg/dL)	5.30 (5.25–5.35)	6.00 (5.95–6.06)	4.52 (4.47–4.57)	<.001
Chronic kidney disease, *n* (%)	7.62 (6.57–8.67)	6.96 (5.90–8.01)	8.35 (6.96–9.74)	.09
eGFR (mL/min/1.73 m^2^)	94.02 (93.30–94.73)	93.49 (92.76–94.21)	94.60 (93.60–95.60)	.03
UACR (mg/g)	5.84 (4.10–9.27)	5.00 (3.64–7.73)	6.82 (4.80–11.06)	<.001

*Note*: All estimates accounted for complex survey designs. For continuous variables: survey‐weighted mean/median (95% CI), *p* value was by weighted linear regression; for categorical variables: weighted percentage (95% CI), *p* value was by weighted Chi‐square test.

Abbreviations: BMI, body mass index; eGFR, estimated glomerular filtration rate; HbA1c, glycated hemoglobin A1c; HDL, high‐density lipoprotein; LDL, low‐density lipoprotein; OGTT, oral glucose tolerance test; UACR, urinary albumin to creatinine ratio.

Overall, the prevalence of hypertension was 30.02% (27.57%, 32.47%) in the whole study population, 23.54% (21.41%, 25.68%) in women and 35.87% (33.54%, 38.20%) in men (Table 2). Compared with women, men had 84% increased risk of prevalence of hypertension. There was an interaction effect of age (<60 years or ≥60 years) on the association between sex (men or women) and prevalence of hypertension (*p* for interaction <.001). Men aged <60 years had higher risk of hypertension than women at the same age (OR, 1.84; 95% CI, 1.59–2.13), while the hypertension risk was attenuated to a level similar between men and women among adults ≥60 years (OR, 1.11; 95% CI, 0.82–1.50) (Table 2).

**Table 2:** Odds ratio (95% CI) of hypertension by sex and age group.

Category	No. of cases/participants/participants	Prevalence (%)	Crude OR (95% CI)	Adjusted OR (95% CI)^a^
Women	783/3160	23.54 (21.41–25.68)	1 [Reference]	1 [Reference]
Men	1491/3927	35.87 (33.54–38.20)	1.82 (1.57–2.10)	1.84 (1.59–2.13)
Age <60 years				
Women	517/2549	19.84 (17.73–21.95)	1 [Reference]	1 [Reference]
Men	1079/3036	34.29 (31.72–36.86)	2.11 (1.79–2.48)	2.11 (1.80–2.48)
Age ≥60 years				
Women	266/611	40.05 (35.26–44.84)	1 [Reference]	1 [Reference]
Men	412/891	43.06 (37.75–48.37)	1.13 (0.83–1.54)	1.11 (0.82–1.50)

^a^ Adjusted for age, race, education, smoking status, alcohol intake, diet quality, physical activity, sleep duration, obesity, diabetes, dyslipidemia, hyperuricemia, and chronic kidney disease. Interactions between age groups and sex in the association with hypertension: *p* < .001.

**Figure 2:**
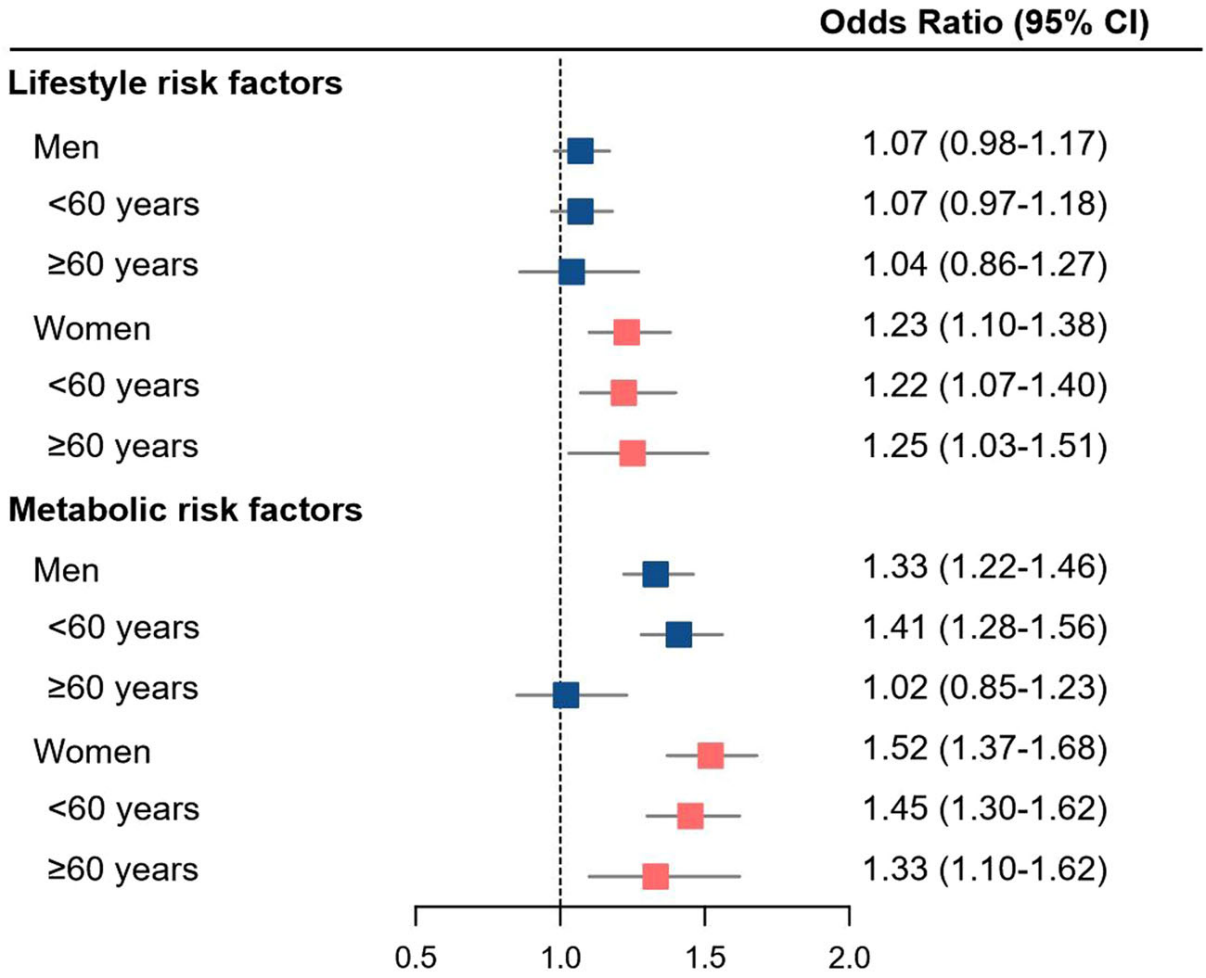
Odds ratios of hypertension associated with per 1‐number increment in lifestyle and metabolic factors. Number of lifestyle and metabolic factors were ranged from 0 to 5, respectively. Models were adjusted for age, race, and education. CI, confidence interval.

Each 1‐increment in numbers of lifestyle risk factors was associated with an increased risk of hypertension in women but not in men (Figure 2). The risks of hypertension were elevated by 33% and 52% with each 1‐increment in numbers of metabolic risk factors in women and men, respectively (Figure 2). Such pattern of association was more evident in men aged <60 years (Figure 2). Sensitivity analysis using multiple imputation of missing data presented a similar association, as compared with those excluded owing to missing lifestyle and metabolic data (Table S2). When we performed a sensitivity analysis using the traditional criteria of hypertension (≥140/90 mmHg), the results did not change materially (Table S3). We also evaluated the associations of individual risk factor with hypertension among participants aged ≥60 or <60 years by sex (Figure S1).

Table 3 showed that for those aged <60 years the combination of lifestyle risk factors accounted for a PAF of 27.2% in men and 48.8% in women. While among those aged ≥60 years, the PAF of lifestyle risk factors was similar between men and women. The combination of metabolic risk factors accounted for a PAF of 37.4% and 38.2% in men and women aged <60 years, respectively. For those aged ≥60 years, metabolic risk factors accounted greater proportion in women (a PAF of 33.0% in women and 14.5% in men). When we investigated the PAF for each risk factor, a greater proportion of the risk for hypertension was explained by lifestyle risk factors such as poor diet in women and in older men. Metabolic risk factors contributed most to hypertension in men aged <60 years, with obesity accounting for a PAF of 20.4%, dyslipidaemia accounting for a PAF of 19.9%, and hyperuricemia accounting for a PAF of 12.9%. Sensitivity analysis using multiple imputation of missing data presented a similar pattern on PAF (Table S4).

**Table 3:** Population attributable fraction for risk of hypertension associated with risk factors by sex and age categories.

Categories	<60 years		≥60 years	
	Men	Women	Men	Women
Lifestyle risk factors				
Current smoking	0	5.6 (2.7 to 8.4)	0	0
Excess alcohol intake	10.2 (3.7 to 16.6)	18.6 (10.4 to 26.6)	5.3 (0.9 to 9.8)	1.5 (−8.3 to 11.2)
Poor diet	13.6 (0.7 to 26.0)	29.9 (20.7 to 38.5)	22.3 (6.4 to 37.0)	21.1 (2.5 to 38.2)
Physical inactivity	0	0	8.2 (−12.8 to 28.5)	0
Unhealthy sleep	9.1 (−0.1 to 18.1)	17.3 (5.4 to 28.8)	0.6 (−14.8 to 16.0)	19.8 (4.4 to 34.2)
Total	27.2 (2.2 to 48.9)	48.8 (22.8 to 68.3)	34.7 (1.8 to 60.9)	35.0 (−1.1 to 63.1)
Metabolic risk factors				
Obesity	20.4 (15.8 to 24.9)	25.7 (19.0 to 32.3)	0	15.0 (9.4 to 20.5)
Diabetes	0	4.5 (−1.8 to 10.8)	4.4 (−3.5 to 12.3)	8.4 (1.0 to 15.6)
Dyslipidaemia	19.9 (7.9 to 31.3)	19.8 (2.6 to 35.9)	0	10.8 (−23.9 to 43.0)
Hyperuricemia	12.9 (9.2 to 16.5)	9.8 (2.2 to 17.3)	5.6 (−2.3 to 13.3)	6.5 (−5.9 to 18.8)
Chronic kidney disease	4.4 (−1.1 to 9.9)	4.3 (−0.1 to 8.8)	8.6 (−7.3 to 24.0)	8.2 (−10.0 to 25.8)
Total	37.4 (16.3 to 55.3)	38.2 (6.7 to 62.7)	14.5 (−15.7 to 42.2)	33.0 (−30.8 to 76.3)

*Note*: Models were adjusted for age, race, and education. The negative population attributable fraction was truncated at the value of point estimate of 0 and individual risk factors with a negative population attributable fraction were not included in the analyses of combination risk factors.

## 4 DISCUSSION

In this study of 7087 U.S. participants, we found that men had 84% increased risk of prevalence of hypertension compared to women. The sex difference in risk for hypertension was more evident in those aged <60 years and lifestyle and metabolic risk factors of hypertension accounted for different proportion between men and women. For those aged <60 years the combination of lifestyle risk factors of hypertension accounted for a greater PAF in women, and the combination of metabolic risk factors accounted for a PAF similarly in men and women. For those aged ≥60 years, the PAF of lifestyle risk factors was similar between men and women and the metabolic risk factors accounted for a greater proportion in women.

Previous studies demonstrated that the association between hypertension with BP cutoff levels of 130/80 mmHg and CVD events was more evident in adults <60 years of age.^26^, ^27^ Consistent with other studies,^3^, ^5^ our study also found that men had a higher risk of hypertension as compared with women in younger population, while there were no differences between men and women in the association with risk of hypertension in older people. The interaction findings between the sex dimorphism in different age group and BP elevation are intriguing, which may be due to the differences in hormonal factors, chromosomal factors, and sex‐biased nonchromosomal gene expression.^2^ The risk conferred by a risk factor (e.g., obesity) in a young adult relative to a young person without the risk factor may be greater than that in an older adult whose risk for hypertension is higher. In addition, the lower PAF of metabolic risk factors in older adults indicated a greater complexity of hypertension risk in older people. Although we accounted for 10 modifiable risk factors in multivariable modeling, unknown or unmeasured age related risk factors not captured in this study were likely to play a role, such as increased oxidative stress.^28^ Furthermore, the age‐related decreases in the impact of metabolic factors in men could be partially influenced by selective survival. Nevertheless, we also observed a similar risk in the impact of lifestyle factors in men according to age, indicating that selective survival is not the only explanation.

The relationship between lifestyle and metabolic risk factors and the risk of hypertension have been identified over the years. Numerous studies have reported that cigarette smoking, excess alcohol consumption, poor diet, lack of physical activity, unhealthy sleep, obesity, diabetes, dyslipidemia, hyperuricemia and CKD were risk factors for elevated BP.^8^, ^10^, ^16^, ^29^, ^30^, ^31^, ^32^ In the Nurses' Health Study, out of six individual modifiable factors including BMI, physical activity, diet, alcohol intake, use of nonnarcotic analgesics, and folic acid supplement, high BMI was the leading contributing risk factor for hypertension (with a PAR% of 40%) in young women.^10^ Despite the well‐established evidence between lifestyle and metabolic risk factors and the risk of hypertension, our study extended previous research by providing a more comprehensive picture of 10 risk factors for hypertension, and by identifying sex differences in risk factors associated with hypertension between adults aged 60 years and older and younger adults. Moreover, based on updated definition of hypertension by ACC/AHA guideline, our analysis provided new insights into the early prevention of hypertension.

Our study has some potential limitations. First, even though we defined hypertension using the newly diagnosed hypertension and excluded previous hypertension, this is a cross‐sectional study and prospective studies are needed to demonstrate establish the causal relation and PAF between these modifiable risk factors and incident hypertension. Second, although using a standard protocol, hypertension was determined by only one attended examination and metabolic measures such as fasting plasma glucose and UACR were tested only once. Measurement errors and a recall bias may exist, and thus some misclassification is possible. These measurement error and recall bias might alleviate the association between hypertension and the risk factors. Third, our results may not be generalizable to populations with other ethnicities.

In conclusion, our results suggested important variation of lifestyle and metabolic risk factors in the contribution of hypertension by sex and age strata. Lifestyle risk factors such as poor diet were mainly responsible for hypertension in men aged ≥60 years and women, while metabolic risk factors such as obesity were the leading causes for hypertension in men aged <60 years. Such findings might be useful in the emerging recognition of the impacts of broad hypertension prevention interventions compared to targeting specific risk factors, and in the understanding with whom to target interventions.

## AUTHOR CONTRIBUTIONS

Jingya Niu, Demin Xu, Jianying Ma, and Shujing Wu conceived and designed the study. Jingya Niu, Jianhong You, and Lixia Suo analyzed the data. Yujie Huang, Mengzhang Wu, Changbin Lu, Xiaodou Niu, and Shujing Wu verified the data. Jingya Niu, Demin Xu, Yujie Huang, and Jianhong You drafted the manuscript. Lixia Suo, Jianying Ma, and Shujing Wu revised the manuscript. All authors contributed to the article and approved the submitted version.

## CONFLICT OF INTEREST STATEMENT

The authors declare no conflict of interest.

## Supporting information

Supporting information.

## Data Availability

The datasets for this study are available on the internet and on easy‐to‐use CD‐ROMs from U.S. National Health and Nutrition Examination Survey (https://www.cdc.gov/nchs/nhanes/, accessed December 15, 2022).
