# Supplementary material for: Sex‐related association of modifiable risk factors with hypertension: A national cross‐sectional study of NHANES 2007–2018
Source: Clin Cardiol. 2023 Oct 5;47(1):e24165. doi: 10.1002/clc.24165 (PMC10768740; doi:10.1002/clc.24165)
Supplement: Supplementary file 1 — Supporting information. [file CLC-47-e24165-s001.doc]

**Sex-Related Association of Modifiable Risk Factors with Hypertension: A National Cross-Sectional Study of NHANES 2007-2018**

**Online Supplement**

Jingya Niu^a,b†^, Demin Xu ^c†^, Yujie Huang^d†^, Jianhong You^e†^, Jie Zhang^f^, Jianan Li^f^, Dan Su^f^, Sanru Lin^f^, Lixia Suo^a*^, Jianying Ma^g,h*^, Shujing Wu^g*^

^†^These authors contributed equally to this work and share first authorship.

**Author Affiliations:**

^a^ Jiading District Central Hospital Affiliated Shanghai University of Medicine and Health Sciences, Shanghai, China

^b^ School of Clinical Medicine, Shanghai University of Medicine and Health Sciences, Shanghai, China

^c^ Department of Cardiac Surgery, Zhongshan Hospital, Fudan University, Shanghai, China

^d^ Medical Department, Zhongshan Hospital (Xiamen), Fudan University, Xiamen, Fujian, China

^e^ Department of Ultrasound, Zhongshan Hospital of Xiamen University, School of Medicine, Xiamen University, Xiamen, Fujian, China

^f^ School of Public Health, Xiamen University, Xiamen, Fujian, China

^g^ Department of Cardiology, Zhongshan Hospital (Xiamen), Fudan University, Xiamen, Fujian, China

^h^ Department of Cardiology, Zhongshan Hospital, Fudan University, Shanghai, China

**Table S1. Prevalence of individual lifestyle and metabolic risk factor by age and sex categories**

| **Categories** | **< 60 years** | |  | **≥ 60 years** | |
| --- | --- | --- | --- | --- | --- |
|  | **Men** | **Women** |  | **Men** | **Women** |
| Lifestyle risk factors |  |  |  |  |  |
| Current smoking | 23.38 (21.24, 25.52) | 18.85 (16.86, 20.83) |  | 14.39 (11.47, 17.32) | 10.88 (8.36, 13.41) |
| Excess alcohol intake | 49.39 (46.25, 52.53) | 59.17 (56.40, 61.95) |  | 19.93 (15.97, 23.89) | 32.74 (28.22, 37.25) |
| Poor diet | 79.61 (77.64, 81.58) | 72.95 (70.30, 75.60) |  | 70.23 (66.11, 74.36) | 59.77 (53.94, 65.59) |
| Physical inactivity | 57.74 (55.16, 60.32) | 76.15 (74.02, 78.28) |  | 71.15 (67.19, 75.10) | 80.29 (75.61, 84.97) |
| Unhealthy sleep | 59.16 (56.45, 61.87) | 57.43 (54.13, 60.74) |  | 57.23 (51.67, 62.80) | 54.03 (47.72, 60.34) |
| Metabolic risk factors |  |  |  |  |  |
| Obesity | 32.46 (30.02, 34.89) | 29.25 (27.02, 31.47) |  | 23.80 (19.95, 27.65) | 26.94 (23.31, 30.56) |
| Diabetes | 7.17 (5.80, 8.53) | 4.92 (3.93, 5.92) |  | 14.18 (11.14, 17.22) | 7.73 (5.24, 10.22) |
| Dyslipidaemia | 68.94 (66.76, 71.13) | 62.82 (60.01, 65.64) |  | 76.79 (72.38, 81.20) | 86.79 (83.32, 90.25) |
| Hyperuricemia | 17.82 (15.90, 19.73) | 11.33 (9.65, 13.02) |  | 14.21 (10.33, 18.09) | 17.11 (13.84, 20.38) |
| Chronic kidney disease | 4.86 (3.83, 5.88) | 6.26 (4.91, 7.61) |  | 16.51 (13.00, 20.03) | 17.69 (14.23, 21.15) |

**Table S2. Odds ratios of hypertension associated with per 1-number increment in lifestyle and metabolic factors by sex and age categories using multiple imputations of missing data**

| **Categories** | **Odds ratio (95% CI)** |
| --- | --- |
| Lifestyle risk factors |  |
| Men |  |
| < 60 years | 1.13 (1.06,1.21) |
| ≥ 60 years | 1.12 (0.97,1.29) |
| Women |  |
| < 60 years | 1.17 (1.08,1.27) |
| ≥ 60 years | 1.17 (1.04,1.32) |
| Metabolic risk factors |  |
| Men |  |
| < 60 years | 1.43 (1.32,1.55) |
| ≥ 60 years | 1.06 (0.95,1.18) |
| Women |  |
| < 60 years | 1.46 (1.35,1.57) |
| ≥ 60 years | 1.19 (1.05,1.35) |

Number of lifestyle and metabolic factors were ranged from 0 to 5, respectively. Models were adjusted for age, race, and education.

Abbreviations: CI, confidence interval.

**Table S3. Odds ratios of hypertension using different criteria associated with per 1-number increment in lifestyle and metabolic factors by sex and age categories**

| **Categories** | **Odds ratio (95% CI)** |
| --- | --- |
| Lifestyle risk factors |  |
| Men |  |
| < 60 years | 1.10 (0.94,1.28) |
| ≥ 60 years | 1.12 (0.87,1.45) |
| Women |  |
| < 60 years | 1.33 (1.08,1.65) |
| ≥ 60 years | 1.31 (1.00,1.72) |
| Metabolic risk factors |  |
| Men |  |
| < 60 years | 1.38 (1.17,1.64) |
| ≥ 60 years | 0.97 (0.77,1.22) |
| Women |  |
| < 60 years | 1.26 (1.01,1.58) |
| ≥ 60 years | 1.42 (1.16,1.85) |

Hypertension was defined as a systolic BP≥140 mmHg or a diastolic BP≥ 90 mmHg. Number of lifestyle and metabolic factors were ranged from 0 to 5, respectively. Models were adjusted for age, race, and education.

Abbreviations: BP, blood pressure; CI, confidence interval.

**Table S4. Population attributable fraction for risk of hypertension associated with risk factors by sex and age categories using multiple imputations of missing data**

| **Categories** | **< 60 years** | |  | **≥ 60 years** | |
| --- | --- | --- | --- | --- | --- |
|  | **Men** | **Women** |  | **Men** | **Women** |
| Lifestyle risk factors |  |  |  |  |  |
| Current smoking | 3.4 (2.1, 4.8) | 1.2 (-0.5, 3.0) |  | 2.6 (1.6, 3.6) | 0 |
| Excess alcohol intake | 8.8 (6.7, 10.9) | 6.4 (4.0, 8.8) |  | 4.8 (4.0, 5.6) | 0.3 (-2.0, 2.5) |
| Poor diet | 14.1 (8.6, 19.5) | 22.2 (17.3, 27.0) |  | 19.4 (12.9, 25.7) | 12.9 (6.3, 19.4) |
| Physical inactivity | 0 | 3.3 (0.9, 5.6) |  | 0 | 0 |
| Unhealthy sleep | 3.2 (0.5, 5.9) | 5.3 (2.3, 8.2) |  | 1.9 (-1.6, 5.4) | 4.6 (0.7, 8.4) |
| Total | 22.3 (13.1, 31.1) | 32.1 (21.9, 41.5) |  | 23.2 (11.4, 34.4) | 16.2 (4.7, 27.3) |
| Metabolic risk factors |  |  |  |  |  |
| Obesity | 20.7 (18.8, 22.5) | 26.2 (23.1, 29.3) |  | 0 | 4.9 (1.8, 7.9) |
| Diabetes | 2.7 (0.4, 5.0) | 5.5 (2.7, 8.3) |  | 0 | 3.2 (0.2, 6.2) |
| Dyslipidaemia | 18.0 (13.1, 22.8) | 15.8 (9.8, 21.7) |  | 0 | 21.3 (12.9, 29.3) |
| Hyperuricemia | 11.1 (10.0, 12.1) | 6.6 (4.2, 9.0) |  | 5.0 (2.0, 8.0) | 0.3 (-2.5, 3.2) |
| Chronic kidney disease | 2.0 (0.5, 3.6) | 4.4 (2.7, 6.2) |  | 3.6 (-0.8, 8.0) | 4.1 (-0.5, 8.8) |
| Total | 35.0 (26.2, 43.1) | 37.1 (25.6, 47.5) |  | 11.3 (3.9, 18.5) | 27.9 (11.8, 42.5) |

Models were adjusted for age, race, and education. The negative population attributable fraction was truncated at the value of point estimate of 0 and individual risk factors with a negative population attributable fraction were not included in the analyses of combination risk factors.

**Figure S1. Odds ratios of hypertension associated with individual lifestyle and metabolic risk factors by sex and age.**

**
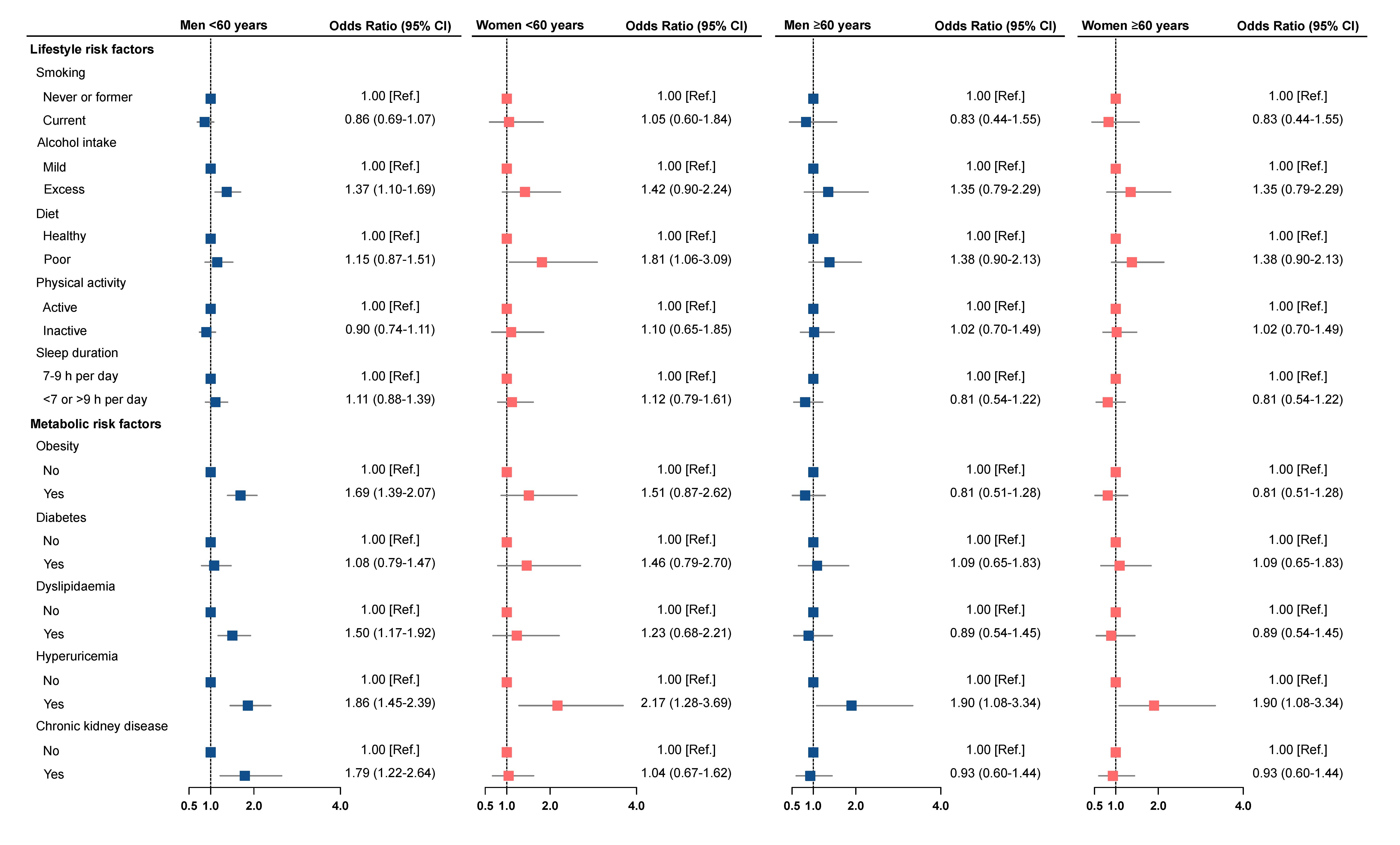
**

Models were adjusted for age, race, and education.

Abbreviations: CI, confidence interval.
